# Pathology of Gall-Stones

**Published:** 1903-10-10

**Authors:** 


### PATHOLOGY OF GALL-STONES.

It is highly probable, in view of the variations in their composition, that gall-stones owe their existence to a variety of causes. It is for future researches to show what are the conditions necessary for the formation of the different kinds of concretions. Cholesterin and bilirubin-calcium are the commonest and most abundant constituents of gall-stones, and a knowledge of these compounds, why they are precipitated from the bile and from whence they are derived, is essential for a proper understanding of the pathology of cholelithiasis. Dr. C. A. Herter[1] summarises our present knowledge on these points. He points out that cholesterin occurs in a lower percentage in the bile obtained from a fistula than in the bile taken from the gall-bladder. The greater amount in the cystic bile can almost certainly, he states, be attributed to cholesterin derived from the epithelial cells lining the gall-bladder. Of the origin of the cholesterin in the hepatic bile little is known. However the derivation of cholesterin from the epithelium of the gall-bladder is of direct practical importance, for it has been shown experimentally that by injecting chemical irritants or the cultures of various bacilli into the gall-bladder, and by this means causing inflammation of the epithelial layer, an excess of cholesterin is formed and added to the bile. This is the first point. Secondly, the bile from gall-bladders containing cholesterin stones is unusually rich in cholesterin. Thirdly, the bile in cases where concretions are present in the majority of cases is found to be infected with micro-organisms. It seems reasonable to suppose, therefore, that cholesterin stones are formed because there is excess of cholesterin in the bile, due to inflammation of the cystic epithelium caused by infection of the gall-bladder by bacteria. With regard to bilirubin-calcium not so much is known. It is a chemical compound in which bilirubin behaves as a monobasic acid. Bilirubin-calcium is insoluble in water, alcohol, chloroform, and ether. There does not seem to be any definite relation between the quantity of calcium and the amount of cholesterin in the bile. Excess of cholesterin with small quantities of calcium is very common, while high calcium concentration with little cholesterin is less common, but is sometimes observed. On the whole it is probable that an excessive concentration of the biliary calcium is answerable for the deposition of the salts of lime, in the form of concretions, and it has been suggested that excessive secretion of mucus is responsible for the calcium concentration. The relation of bacteria to cholelithiasis has been demonstrated both by Gilbert and Mignot, in the laboratory, by introducing bacterial cultures into the gall-bladders of animals (dogs and rabbits). By these means gall-stones have been artificially produced. It was found necessary in these experiments to use attenuated cultures, and the best results were obtained with those bacteria which had previously been cultivated in diluted bile. These experiments have shown that the following organisms are capable of producing cholelithiasis:—Bacillus coli, bacillus typhosus, staphylococcus pyogenes, and streptococcus pyogenes.

[1] Medical News, Sept. 19th.

